# Dissecting the human serum antibody response to secondary dengue virus infections

**DOI:** 10.1371/journal.pntd.0005554

**Published:** 2017-05-15

**Authors:** Bhumi Patel, Patti Longo, Michael J. Miley, Magelda Montoya, Eva Harris, Aravinda M. de Silva

**Affiliations:** 1 Department of Microbiology and Immunology, University of North Carolina School of Medicine, Chapel Hill, North Carolina, United States of America; 2 Department of Pharmacology, University of North Carolina School of Medicine, Chapel Hill, North Carolina, United States of America; 3 Division of Infectious Diseases and Vaccinology, School of Public Health, University of California, Berkeley, California, United States of America; University of California, Davis, UNITED STATES

## Abstract

Dengue viruses (DENVs) are mosquito-borne flaviviruses and the causative agents of dengue fever and dengue hemorrhagic fever. As there are four serotypes of DENV (DENV1-4), people can be infected multiple times, each time with a new serotype. Primary infections stimulate antibodies that mainly neutralize the serotype of infection (type-specific), whereas secondary infections stimulate responses that cross-neutralize 2 or more serotypes. Previous studies have demonstrated that neutralizing antibodies induced by primary infections recognize tertiary and quaternary structure epitopes on the viral envelope (E) protein that are unique to each serotype. The goal of the current study was to determine the properties of neutralizing antibodies induced after secondary infection with a different (heterotypic) DENV serotypes. We evaluated whether polyclonal neutralizing antibody responses after secondary infections consist of distinct populations of type-specific antibodies to each serotype encountered or a new population of broadly cross-neutralizing antibodies. We observed two types of responses: in some individuals exposed to secondary infections, DENV neutralization was dominated by cross-reactive antibodies, whereas in other individuals both type-specific and cross-reactive antibodies contributed to neutralization. To better understand the origins of type-specific and cross-reactive neutralizing antibodies, we analyzed sera from individuals with well-documented sequential infections with two DENV serotypes only. These individuals had both type-specific and cross-reactive neutralizing antibodies to the 2 serotypes responsible for infection and only cross-reactive neutralizing antibodies to other serotypes. Collectively, the results demonstrate that the quality of neutralizing (and presumably protective) antibodies are different in individuals depending on the number of previous exposures to different DENV serotypes. We propose a model in which low affinity, cross-reactive antibody secreting B-cell clones induced by primary exposure evolve during each secondary infection to secrete higher affinity and more broadly neutralizing antibodies.

## Author summary

The four dengue virus serotypes are emerging mosquito-borne flaviviruses and the causative agents of dengue fever and dengue hemorrhagic fever/dengue shock syndrome. Infected people develop protective immunity to the infecting serotype but remain susceptible to secondary infections with new serotypes. Both antibodies and T-cells are responsible for protection against re-infection by the same serotype. The goal of the current study was to analyze the properties of antibodies in people who have been exposed to a single or secondary dengue virus infections. We found that people exposed to a single infection have neutralizing antibodies that mainly bind sites that are unique to the infecting serotype. In contrast, secondary infections induced more complex mixtures of neutralizing antibodies that target regions that unique to each serotype and regions that are conserved between serotypes. Our results have implications for understanding protective responses after natural infections as well as responses induced by dengue vaccines.

## Introduction

Dengue Virus (DENV) is a mosquito-borne *flavivirus* and the causative agent of dengue fever and dengue hemorrhagic fever (DHF) [1]. Several hundred million people are estimated to acquire DENV infections each year[2]. Dengue infections can be clinically inapparent or lead to symptoms that range from an undifferentiated fever to severe DHF and dengue shock syndrome (reviewed in [3, 4]).

The DENV complex consists of 4 viruses designated as serotypes (DENV1-4)[1]. Primary infection by DENV leads to long-term protection against the serotype of infection (homologous serotype) but not other serotypes (heterologous serotypes) [5–7]. Subsequent secondary infection with a new serotype results in serotype cross-neutralizing antibodies that correlate with durable protection against 2 or more serotypes [5, 7]. Recent studies have defined the properties of human antibodies responsible for serotype-specific neutralization after primary infection [8–12]. In the present study we investigated the properties of serum neutralizing antibodies produced after recovery from secondary DENV infections.

The DENV envelope (E) protein, which binds to cellular receptors and mediates viral entry and fusion, is the main target of neutralizing and protective antibodies [13]. The ectodomain of E protein is composed of three domains: I, II and III (EDI, EDII and EDIII)[14]. Each DENV particle has 180 monomers of E that are organized into 90 dimers that cover the entire surface of the virus. The E proteins are arranged with icosahedral symmetry with each asymmetric unit containing portions of three homodimers[15]. After primary DENV infection, people develop a mix of DENV serotype cross-reactive and type-specific antibodies. The cross-reactive antibodies are weakly neutralizing and have been implicated in antibody dependent enhanement of DENVs during secondary infections [8, 16–18]. The serotype-specific antibodies are strongly neutralizing and, presumably, responsible for long-term protection aganst re-infection with the same serotype. Quaternary epitopes formed after assembly of E molecules into higher order structures required for virion assembly are major targets of type-specific neutralizing antibodies [8–12].

Here we report on the properties of serum neutralizing antibodies in people exposed to 2 or more DENV infections. The studies were designed to test if people exposed to secondary infections have neutralizing antibodies that mainly recognize epitopes that are conserved between serotypes or epitopes that are unique to each serotype previously encountered. Unlike primary DENV infections that result in predominantly serotype-specific polyclonal neutralizing antibody responses, some people exposed to secondary infections had neutralizing antibodies that mainly recognized epitopes conserved between serotypes, while others had antibodies that targeted both type-specific and conserved epitopes.

## Materials and methods

### Ethics statement

Blood donations were obtained from individuals who had traveled to dengue-endemic regions and experienced a primary DENV infection. These human samples were obtained with informed consent approximately 2 to 10 years after DENV infection. All blood donations were collected in compliance with the Institutional Review Board of the University of North Carolina at Chapel Hill. Written informed consent was obtained from all subjects before participation in the study. Blood donations were obtained from individuals who had traveled to dengue-endemic regions or people living in endemic areas. All blood donations were collected in compliance with the Institutional Review Board of the University of North Carolina at Chapel Hill and the University of California at Berkeley. Written informed consent was obtained from all subjects before participation in the study.

### Immune sera

#### Dengue traveler study

Human sera were collected from individuals who had experienced a DENV infection during travel to a DENV endemic area (Table 1). All donations were collected in compliance with the Institutional Review Board of the University of North Carolina at Chapel Hill (Protocol #08–0895). Samples were classified as primary infections if they had neutralizing antibody to only one serotype (≥ 1:40 Neut_50_ titers) or a titer that is 4-fold higher for one DENV serotype compared to low titers to the other serotypes. Samples were classified as secondary infections if they had Neut_50_ titers greater than a 1:40 dilution for two or more DENV serotypes.

**Table 1:** Panel of naïve and late convalescent DENV-immune human sera.

				Reciprocal of Neut_50_ Titer				
Donor ID	Location of infection	Year of most recent known infection	Approximate interval between infection and sampling (years)	DENV1	DENV2	DENV3	DENV4	Sero Status^†^
**30**	-	-	-	<20	<20	<20	<20	Naïve
**1**	Sri Lanka	1996	9	50	443	70	43	Primary DENV2
**110**	Kuala Lumpur	1998	10	205	1280	153	96	Primary DENV2
**0**	Indian Subcontinent	Unknown	Many	304	630	452	105	Repeat
**25**	Philippines	1975	25	179	233	47	71	Repeat
**27**	Thailand/Cambodian border	1981	25	878	249	218	71	Repeat
**121**	Philipines	1995	16	92	371	325	179	Repeat
**130**	French Polynesia	1989	25	674	347	86	62	Repeat

^†^ Primary defined as reciprocal Neut_50_ titer greater than 40 for one serotype only or a reciprocal Neut_50_ titer that is at least 4-fold greater for one serotype. Secondary defined as reciprocal Neut_50_ titer greater than 40 for two or more serotypes and the highest titer not 4-fold greater than the 2^nd^ highest titer.

#### Nicaraguan Pediatric Dengue Cohort Study

The Nicaraguan Pediatric Dengue Cohort Study is an ongoing community-based prospective study of children 2–14 years old established in 2004 in Managua, Nicaragua [19, 20]. Participants are encouraged to present at the first sign of illness to the HCSFV, where study physicians provide medical care and screen for signs and symptoms of dengue. Suspected dengue cases, as defined by the traditional WHO dengue case definition (WHO Guidelines 1997) and febrile participants without other apparent origin (undifferentiated febrile illnesses) are analyzed for DENV infection using serological, molecular and virological methods in acute-phase (days 1–5) and convalescent-phase (14–21 days post-onset of fever) blood samples [19, 20]. Healthy annual blood samples are also collected for serological analysis. Neut_50_ titers were determined in annual samples using a flow cytometry-based assay with reporter viral particles representing the four DENV serotypes in human monocytic U937 cells expressing the DENV attachment factor DC-SIGN[21, 22]. The PDCS was approved by the Institutional Review Boards of the University of California, Berkeley, and the Nicaraguan Ministry of Health. Parents or legal guardians of all subjects provided written informed consent, and subjects 6 years of age and older provided assent.

### Virus and recombinant envelope protein

WHO reference strains, DENV1 (West Pac 74), DENV2 (S-16803), DENV3 (CH-53489) and DENV4 (TVP-376) were used in the present study. The strains were grown in C6/36 mosquito cells to generate infectious stocks and Vero-81 mammalian cells to generate purified antigen. DENVs from culture media were purified by density gradient and ultracentrifugation as previously described [23]. DENV2 (NGC) purified live virion antigen was purchased from Microbix Biosystems, Inc. (Mississauga, Ontario, Canada). DENV2 envelope protein was produced as a soluble recombinant protein (amino acids 1–397 of S-16803) using the Bac-to-Bac Baculovirus Expression System (Invitrogen Life Technologies) and Sf9 insect cells. The protein was purified from cell culture supernatant using an antibody column coated with 4G2, a serotype-cross reactive mouse monoclonal that targets a conserved region on the envelope protein. Structure of the recombinant protein was verified by ELISA using monoclonal antibodies that bind to well-defined epitopes on all three domains of E protein.

### Fluorescent Activated Cell Sorting (FACS)-based DENV neutralization assay

DENV neutralizing activity of human immune sera was assessed using a flow cytometry-based assay with U937 human monocytic cells stably transfected with DC-SIGN as previously described [24]. In brief, serially diluted human sera were incubated with virus for 45 min at 37°C followed by the addition of U937 DC-SIGN cells. Cells were incubated with virus for 2 hours at 37°C, washed with media to remove immune sera and unbound virus and incubated for 24 hours at 37°C. Cells were fixed, permeabilized and stained with 2H2-Alexa Flour 488, a mouse monoclonal that binds to DENV pre-membrane protein. Infected cells were quantified using a Guava flow-cytometer (Milipore). Stained cells were analyzed using GraphPad Prism version 6.00 (La Jolla California USA, www.graphpad.com) to calculate 50% neutralization titers as previously described [25]. We determined if depletion of specific antibodies resulted in statistically significant (P<0.05) changes in Neut_50_ values by comparing neutralization curves using the Extra Sum of Squares F test (Non-linear regression: Compare function in GraphPad Prism version 6.00). Each control and antigen depleted serum sample was tested in a single neutralization assay in duplicate using all 4 DENV serotypes.

### Detection of DENV binding antibodies

ELISAs were conducted for both confirmation of depletion and the assessment of binding activity to all four DENV serotypes following depletion of human DENV immune sera. Plates were coated with either 50 ng of purified DENV or 100 ng of DENV rE in carbonate buffer at pH 9.6 for 2 hours at room temperature. The plates were blocked with Tris-buffered Saline containing 0.05% Tween 20 with 3% Normal Goat Serum followed by an incubation with a 1:40 dilution of control or DENV depleted human sera for 1 hour at 37°C. Alkaline phosphatase conjugated Goat anti-human IgG (Sigma) was added to the plates for 1 hour at 37°C. The plates were washed and then developed by adding p-nitrophenyl phosphate substrate. The optical density at 405nm was recorded using a spectrophotometer.

### Whole virus depletion of DENV-specific antibodies from human immune sera

Purified DENV was absorbed onto 4.5-μm Polybead polystyrene microspheres (Polysciences, Inc.) at a bead (ul) to ligand (ug) ratio of 5:2. Beads were washed three times with 0.1M Borate buffer (pH 8.5) followed by an overnight incubation with purified DENV and 0.1M Borate buffer (pH 8.5) at room temperature (RT). Control beads were incubated with an equivalent amount of BSA. The control and virus absorbed beads were then blocked with a 10 mg/ml BSA solution for 30 minutes at RT followed by four washes with PBS. DENV-specific antibodies were depleted from human sera by incubating virus absorbed beads with human sera diluted 1:10 in PBS for 45 minutes at 37°C. Typically three cycles were performed to remove dengue-specific antibodies in each serum sample. Successful depletion of DENV-specific antibodies was confirmed via an ELISA with purified DENV coated plates.

### Recombinant E protein depletion of DENV-specific antibodies from immune sera

Purified DENV2 rE protein was conjugated to magnetic dynabeads M-270 Epoxy (Invitrogen by Life Technologies) with a bead (mg) to ligand (ug) ratio of 5:1. Beads were washed three times with 0.1M Sodium Phosphate (7.4) followed by an overnight incubation with equal volumes of purified rE protein, 0.1M Borate buffer (pH 9.5) and 3M Ammonium sulfate at 37°C. Control beads were incubated with the equivalent amount of BSA. Control and DENV2 rE conjugated beads were then blocked by incubating with a 10 mg/ml BSA solution followed by four washes with PBS. DENV2 rE-specific antibodies were depleted from human sera by incubating rE conjugated beads with human sera diluted 1:10 in PBS for 45 minutes at 37°C three times with end-over-end mixing. Successful depletion of DENV2 rE-specific antibodies was confirmed via an ELISA with purified DENV2 rE coated plates.

### Calculating the proportion of re binding neutralizing antibodies

After depletion of sera using DENV2 rE antigen, the percentage of rE binding DENV2 neutralizing Abs were calculated using the following formula.

$$\begin{array}{l} \%\text{ rE binding DENV}2\text{ neutralizing Abs}\\ \begin{array}{cc} & \text{= (1- }\left({\text{Neut}}_{50}\text{ after DV}2\text{ rE deletion / }\left({\text{Neut}}_{50}\text{ after control deletion}\right)\right)*100. \end{array} \end{array}$$

## Results

To characterize neutralizing antibodies in DENV immune sera, we initially used a panel of eight human DENV immune sera from people who had been exposed to DENV during travel or previous residency in a dengue endemic area (Table 1). The samples were collected between 9 and 30 years of their most likely exposure to DENV. Two sera are from primary DENV2 cases while the rest are from people exposed to two or more infections. A serum sample from a dengue naïve human donor was used as a negative control.

To determine if DENV serotype cross-reactive or type-specific antibodies were responsible for neutralizing activity in each serum sample, we used polystyrene beads coated with purified dengue virions to deplete different populations of antibodies from serum samples. The antibodies in each serum sample were depleted using beads coated with purified DENV2 or an equal mixture of DENV1, 3 and 4. As a control, the serum was also incubated with beads coated with BSA. The DENV2 depletions were expected to remove DENV2 type-specific and DENV serotype cross-reactive antibodies, while retaining any DENV1, 3 or 4 type-specific antibodies in the sample. After depleting with DENV2, any reduction in DENV1, 3 and 4 neutralizing activity in the sample was attributed to cross-reactive (heterotypic) antibodies. Similarly, when serum samples were depleted with beads containing equal amounts of purified DENV1, 3 and 4, any reduction in DENV2 neutralizing antibody was attributed to cross-reactive antibodies.

### Depletion of DENV binding antibodies from primary DENV2 immune sera

**Fig 1:**
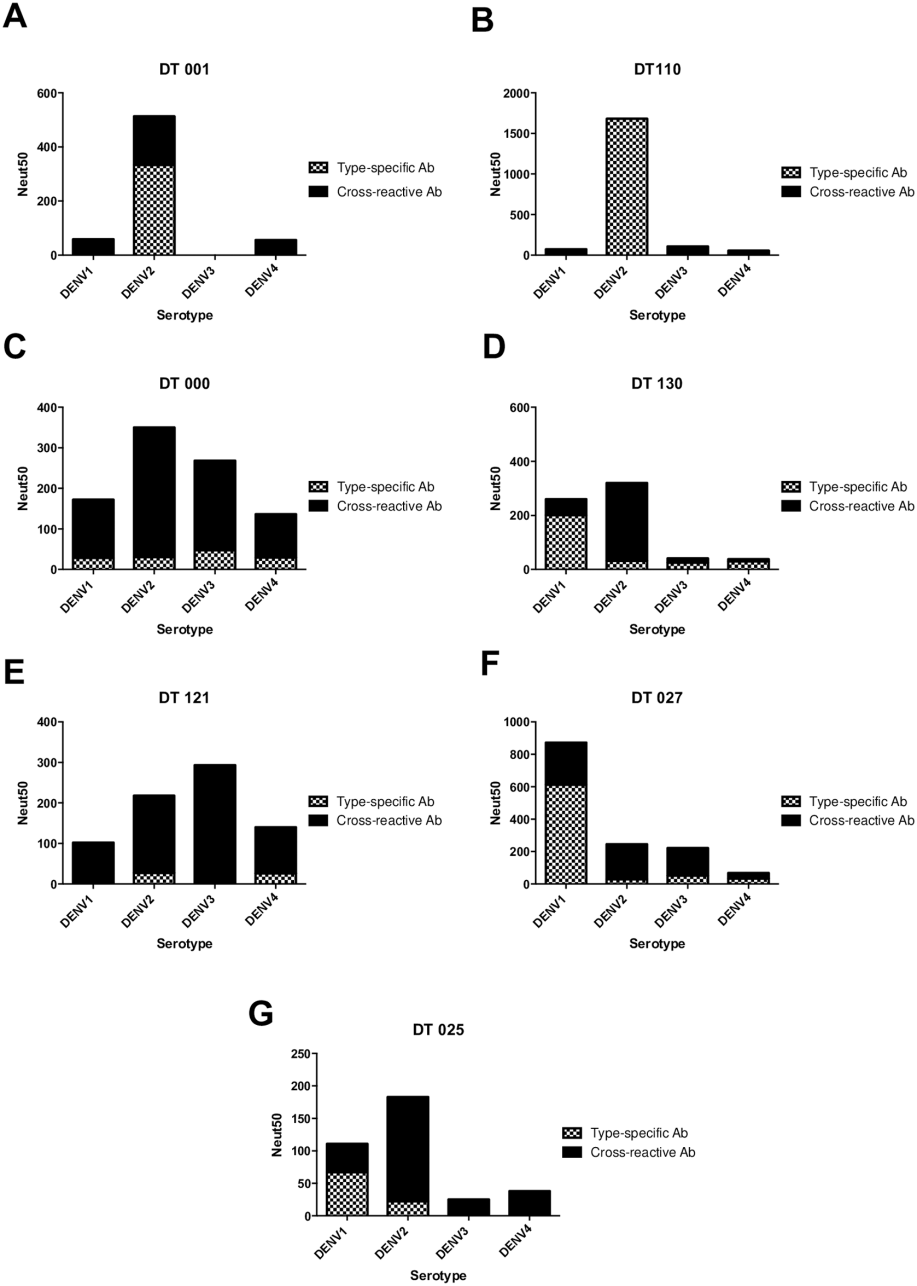
Neutralization properties of primary and secondary infection DENV-immune human sera depleted of DENV-specific antibodies. Polystyrene beads coated with either purified DENV2 or a mixture of purified DENV1, 3 and 4 were used to deplete DENV2 primary immune sera (DT001 and DT110) and DENV secondary infection immune sera (DT000, DT130, DT121, DT027 and DT025) of DENV-specific antibodies. After confirming depletion of relevant antibodies (see S1 and S2 Figs), sera was tested for neutralization of DENV1-4. **A-B**. Levels of type-specific and cross-reactive neutralizing antibodies in 2 individuals exposed to primary DENV2 infections. **C-G**. Levels of type-specific and cross-reactive neutralizing antibodies in individuals exposed to secondary DENV infections.

Depletion of two primary DENV2-immune sera (DT001 and DT110) using beads coated with DENV2 resulted in a reduction of binding to DENV2 as well as DENV1, 3 and 4, demonstrating removal of cross-reactive and type-specific antibodies in the sample (S1 Fig). Antibody depletion with the homologous (serotype of infection) DENV2 antigen led to a significant (P<0.05; Extra Sum of Squares F test to compare neutralization curves) drop in DENV2 neutralization (S1 Fig). Following depletion with DENV1, 3 and 4 coated beads, ELISA confirmed removal of cross-reactive antibodies, while still retaining DENV2 type-specific antibodies in the sample (S1 Fig). After removal of cross-reactive antibodies, the samples retained most of the DENV2 neutralizing activity and the neutralization titers of the control and depleted samples were not significantly different (P>0.05; Extra Sum of Squares F test to compare neutralization curves) (S1 Fig). Fig 1A and 1B). These results are in agreement with previous reports that following primary DENV infections neutralization of the homologous serotype is mainly mediated by type-specific antibodies [8].

### Depletion of DENV binding antibodies from secondary infection DENV immune sera

Following secondary DENV infections, individuals are, typically, protected from two or more serotypes. We tested whether individuals exposed to secondary infections generate individual type-specific neutralizing populations of antibodies against each serotype or a broadly neutralizing cross-reactive population against 2 or more serotypes. We depleted secondary infection sera using beads coated with DENV2 or a mixture of DENV1, 3 and 4 and performed neutralization assays (S2 Fig). In some individuals DENVs were almost exclusively neutralized by cross-reactive antibodies, while others had a mixture of cross-reactive and type-specific neutralizing antibodies (Fig 1C, 1D, 1E, 1F and 1G). For example, subject DT000 had high levels of neutralizing antibodies to all 4 serotypes (Table 1). When DENV2 binding antibodies were depleted from the DT000 sample, there was a significant loss (P<0.05; Extra Sum of Squares F test) of DENV2 neutralization as expected (S2 Fig). However, there was also a significant loss (P<0.05; Extra Sum of Squares F test) of DENV1, 3 and 4 neutralization (S2 Fig) indicating that these serotypes were neutralized by cross-reactive antibodies (Fig 1C). Reciprocal depletion of DT000 immune sera with DENV1, 3 and 4 antigens resulted in a significant loss (P<0.05; Extra Sum of Squares F test) of DENV2 neutralization, indicating that cross-reactive antibodies were responsible for neutralization of DENV2 as well (Fig 1C and S2 Fig). Subject DT121, another subject with neutralizing antibodies to all 4 serotypes, also had a response that was dominated by cross reactive neutralizing antibodies (Fig 1E and S2 Fig).

DT130, a subject that strongly neutralized DENV1 and 2 but not 3 and 4 (Table 1), had a mixture of type-specific and cross-reactive neutralizing antibodies (Fig 1D). Following removal of DENV2 binding antibodies, we observed a major loss (P<0.05; Extra Sum of Squares F test) of DENV2 neutralization and only a partial loss of DENV1 neutralization (S2 Fig). This result indicates that both type-specific and cross reactive antibodies are responsible for the high DENV1 neutralizing activity in this individual (Fig 1D). Reciprocal depletion with DENV1, 3 and 4 antigens removed all the DENV2 neutralizing activity demonstrating that cross-reactive antibodies were responsible for neutralization (Fig 1D and S2 Fig). Samples DT025 and DT027 also exhibited a similar pattern in which both type-specific and cross-reactive antibodies contributed to DENV neutralization (Fig 1F and 1G, and S2 Fig). Unlike primary DENV infections that stimulate durable serotype-specific neutralizing antibody responses, we conclude that secondary infections result in more complex mixtures of neutralizing antibodies that recognize serotype-specific and cross-reactive epitopes. The proportions of these two classes of antibodies varied between individuals exposed to secondary infections.

### Depletion of DENV binding antibodies from individuals exposed to a known sequence infection with two different DENV serotypes

To better understand different patterns of type-specific and cross reactive neutralizing antibodies in people exposed to secondary DENV infections, we analyzed serum samples from 3 individuals with well documented histories of two sequential infections with different serotypes of DENV (Table 2). These samples were obtained from a long-term prospective pediatric DENV cohort study in Nicaragua [22]. Two of the subjects had been exposed to a first DENV2 infection followed by a second DENV3 infection (Subjects 985.8 and 3428.8). One subject had been exposed to a DENV1 infection followed by a DENV3 infection (Subject 2934.7). In sera collected several months after the second infection, all three subjects had varying levels of neutralizing antibodies to at least 3 different serotypes (Table 2).

**Table 2:** Panel of Nicaragua pediatric cohort human immune sera used in the study.

				Reciprocal of Neut_50_ Titer following second infection			
Donor ID	Serotype/Year of primary infection	Serotype/Year of second infection	Year sample collected	DENV1	DENV2	DENV3	DENV4
**985.8**	DENV2/2005	DENV3/2010	2011	304	1586	887	<10
**2934.7**	DENV1/2005	DENV3/2009	2010	85	21	154	45
**3428.8**	DENV2/2005	DENV3/2011	2011	105	2143	2456	467

**Fig 2:**
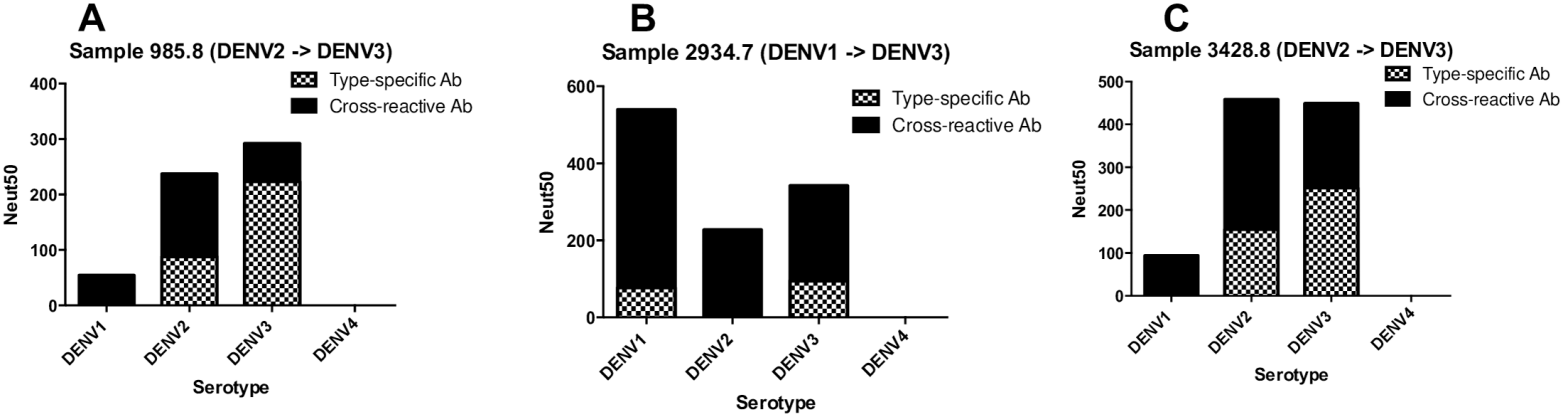
Properties of DENV neutralizing antibodies after sequential infections with two different DENV serotypes. Subjects 985.8 **(A)** and 3428.8 **(C)** experienced sequential DENV2 → DENV3 infections. Subject 2934.7 **(B)** experienced sequential DENV1 → DENV3 infections. Polystyrene beads coated with DENV serotypes that infected each individual were used to deplete different populations of antibodies in the samples. After confirming depletion of relevant antibodies, the sera were tested for neutralization of DENV1-4 (see S3 Fig). The graphs depict the levels of DENV serotype-specific and cross-reactive neutralizing antibodies in each subject after the second infection.

We depleted each post-second infection sample using beads coated with the DENV serotype of the 1^st^ or 2^nd^ infection and performed neutralization assays to estimate levels of cross-reactive and type-specific neutralizing antibodies against each DENV serotype. All three subjects had a mixture of type-specific and cross-reactive neutralizing antibodies directed to the serotypes of known 1^st^ and 2^nd^ infection (Fig 2 and S3 Fig). For serotypes not “seen” by the individual, neutralization was driven by cross-reactive antibodies only (Fig 2 and S3 Fig).

### Depletion of DENV recombinant E protein binding antibodies

**Fig 3:**
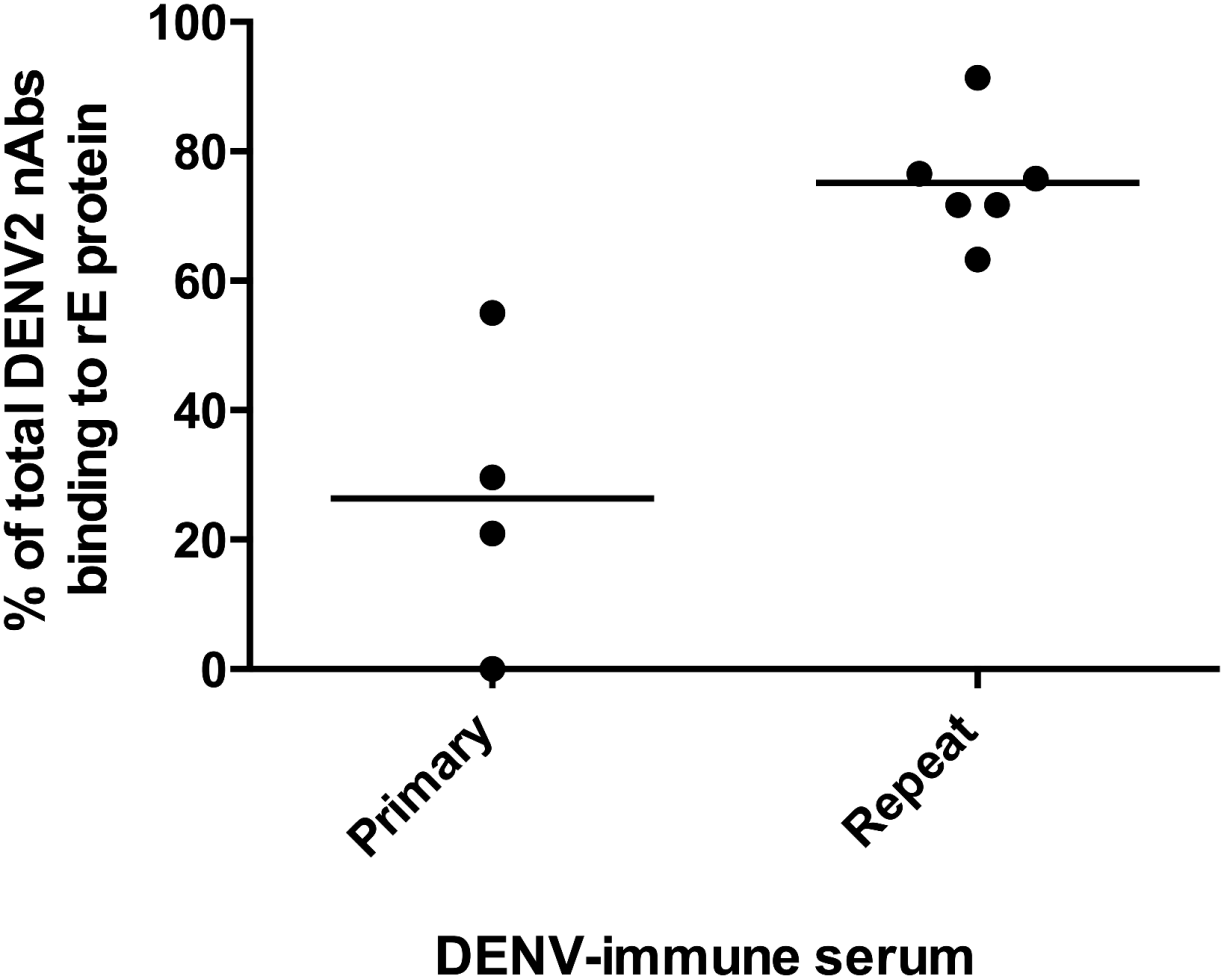
People exposed to secondary DENV infections develop neutralizing antibodies that target simple epitopes on recombinant E protein. Primary DENV2 or secondary DENV infection convalescent immune sera depleted of rE protein binding antibodies were assessed for neutralization of DENV2. In people exposed to secondary DENV infections, a greater proportion of DENV2 neutralizing antibodies bound to epitopes displayed on rE protein compared to people exposed to primary DENV2 infections (Mean of 75% vs. 26%; *P<0.018 unpaired Student t Test to compare means). The primary immune sera data are from 2 subjects analyzed in this study (see S4 Fig) and two subjects analyzed in a previous study[8]. All the secondary infection data are from samples analyzed for current study.

Our previous studies with primary DENV immune sera revealed that type-specific neutralizing antibodies mostly targeted quaternary epitopes expressed on E protein dimers or higher order structures but not recombinant E protein (rE), which is mainly a monomer in solution[8]. We, next, addressed if neutralizing antibodies induced by secondary DENV infections targeted simple or quaternary epitopes on E protein. When convalescent immune sera from individuals exposed to primary DENV2 infections were depleted of DENV2 rE binding antibodies, we observed no significant loss of DENV2 neutralization (P>0.05; Extra Sum of Squares F test), confirming the importance of quaternary structure neutralizing antibody epitopes after primary infection (Fig 3 and S4 Fig). When convalescent immune sera from individuals exposed to secondary DENV infections (Table 1) were depleted of DENV2 rE binding antibodies and tested for DENV neutralization, we observed a significant decrease in DENV2 neutralizing antibody titers (P<0.05; Extra Sum of Squares F test) (Fig 3 and S5 Fig). Our results indicate that epitopes displayed on rE protein are a major target of DENV2 neutralizing antibodies after secondary infections but not primary DENV2 infections.

## Discussion

Recently several studies have described the properties of neutralizing antibodies generated following primary DENV infections and the epitopes targeted by these antibodies [8–10, 18, 26]. Although primary infections stimulate both DENV serotype-specific and cross reactive binding antibodies, only the type-specific antibodies have been linked to durable neutralizing and protective responses. These type-specific antibodies target tertiary and quaternary structure E protein epitopes displayed on the surface of the virus [8–10, 12].

In the current study we observed that the majority of neutralizing antibodies that develop after secondary DENV infections recognize serotype cross reactive epitopes. The cross-reactive neutralizing antibodies bound to simple epitopes on soluble E protein as well as more complex epitopes displayed on the intact DENV particle. Indeed, serotype cross-reactive and neutralizing human monoclonal antibodies isolated from people exposed to secondary DENV infections bind with high affinity to simple epitopes on domain II as well as quaternary epitopes that span across two E proteins forming a single dimer [16, 27–29].

Following secondary infections, some people had a single population of DENV serotype cross-reactive and cross-neutralizing antibodies, whereas others had a mixture of type-specific and cross-reactive neutralizing antibodies. We suspect that subjects like DT000 and 121 who mainly had cross-reactive neutralizing antibodies are likely to have had multiple DENV exposures resulting in broad neutralization of all 4 serotypes. Subjects like DT27 and 130, who had a mixture of type-specific and cross-reactive neutralizing antibodies are likely to represent people who have been exposed to sequential infections with 2 serotypes only. Indeed, when we tested samples from three subjects enrolled in a long-term prospective pediatric cohort study in Nicaragua with know exposure to just two sequential DENV serotype infections, all three subjects had a mixture of type-specific and cross reactive neutralizing antibodies to the serotypes responsible for the first and second infections. Tsai and colleagues recently characterized DENV neutralizing antibodies in volunteers infected with a single serotype monovalent live attenuated DENV vaccine or sequentially infected with two monovalent DENV vaccines representing different serotypes [30]. They also observed type-specific neutralizing antibodies in volunteers who received the single serotype vaccine and mixture of type-specific and cross reactive neutralizing antibodies after sequential infection with 2 different serotypes.

**Fig 4:**
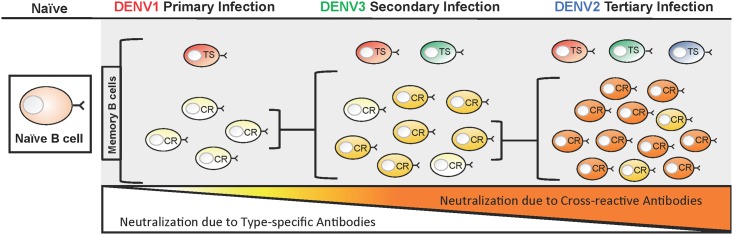
Model of B cell maturation following sequential DENV infections. With each successive DENV infection, the ratio of serotype-specific (TS) and cross-reactive (CR) antibodies that contribute to DENV neutralization changes. During a primary infection (DENV1 in this example), dengue-specific naïve B cells are activated and these cells give rise to both memory B cells (MBCs) and antibody secreting long lived plasma cells (LLPCs). This primary response is dominated MBC and LLPCs clones producing low affinity, weakly neutralizing serotype CR antibodies. The primary response also contains rare MBC and LLPCs producing TS antibodies that strongly neutralize DENV1. Following a secondary infection with a new serotype (DENV3 in this example), the overall DENV-specific B-cell response will be dominated by the activation and expansion of DENV1 and 3 cross-reactive MBCs induced by the primary infection. MBCs producing CR antibodies that bind to the second infecting serotype with high affinity will be preferentially activated. These activated cells will re-enter germinal centers and undergo further rounds of somatic hyper mutation. CR B-cells with high affinity for the second serotype will be selectively expanded to give rise to cross-reactive MBC and LLPCs that strongly cross-neutralize multiple serotypes. In the figure this increase in affinity and neutralization is depicted by an increase in the color gradient (light yellow to bright orange) of CR B-cells. Following a tertiary infection (DENV2 in this example), this process is secondaryed again and results in a population of CR MBCs and LLPCs that dominate the neutralizing antibody response. While the B-cell clones producing TS strongly neutralizing antibodies are also likely to be maintained through each successive round of infection, the TS response will account for only a small fraction of the total neutralizing response.

We propose that low affinity DENV cross reactive memory B-cells derived from primary infections undergo antibody somatic hyper mutation and each subsequent DENV exposure selects and expands rare affinity matured clones with greater neutralization breadth and potency (Fig 4). This model is supported by studies comparing the avidity and neutralization of both monoclonal antibodies and polyclonal sera from people after primary and secondary DENV infections. Analysis of polyclonal human sera following DENV infections revealed that the avidity of DENV antibodies following secondary infection was higher than that of antibodies generated following a primary infection [31]. In agreement with this, studies focusing on group-reactive MAbs derived from primary and secondary DENV infected patients found that the group-reactive MAbs from patients with secondary infection had stronger neutralization potencies and higher binding avidities than those derived from patients with primary infection [29, 32, 33]. Additional studies have identified a class of broadly neutralizing human antibodies produced by plasmablasts in hospitalized cases of secondary DENV infections. Structural analysis of these broadly neutralizing antibodies in complex with rE revealed that these antibodies recognize serotype invariant sites at the E dimer interface. Collectively, these studies support the idea that low affinity, weakly neutralizing antibody clones generated followed primary DENV infections give rise to antibodies of increasing breadth and neutralization potency with each subsequent exposure (Fig 4).

In this study we analyzed in-depth convalescent blood samples from 10 individuals exposed to DENV infections. While the small sample size is a weakness, it is challenging to perform antibody depletion studies on larger panels because of the complexity of the studies and the volume of blood required. Another limitation of our study is that the infection history of some of the study subjects was inferred by the neutralizing antibody profile and travel history, therefore, definite conclusions relating antibody population characteristics to the number of secondary infections cannot be made. However, three subjects with known sequences of two DENV infections support our conclusion that sequential infections with two serotypes result in a mixture of cross-reactive and type-specific neutralizing antibodies to serotypes responsible for infections, while inducing cross-reactive neutralizing antibodies only to the serotypes not “seen” by the host. Studies are currently in progress using additional samples from human dengue cohort studies with well-defined infection histories to further test our model about the roles of sequential infections and antibody somatic hypermutations in cross-protective immunity.

Our studies show that the prior DENV immune status of an individual has a profound effect on the quality of neutralizing antibodies that develop after an infection. These findings are relevant to the development of live attenuated dengue vaccines that strive to provide simultaneous protection against all four serotypes. In DENV naïve individuals who are vaccinated with a single dose of live attenuated DENV vaccines, protection is likely to require type-specific protective antibody responses to each serotype. In the case of tetravalent live DENV vaccine formulations, these responses may not be balanced towards each serotype [34, 35]. On the other hand, DENV-immune individuals receiving a tetravalent vaccine are likely to generate broadly neutralizing and protective responses even if individual components in the vaccine perform poorly. Indeed, a recent live attenuated tetravalent dengue vaccine efficacy trial demonstrated higher efficacy in dengue pre-immune individuals compared to DENV naïve individuals [36, 37]. Overall, it is clear that a better understanding of the antibody response transition from primary to secondary infection is needed to understand and improve the performance of dengue vaccine in the current pipeline.

## Supporting information

S1 Fig Binding and neutralization properties of primary infection DENV2-immune human sera following depletion of DENV-binding antibodies. Polystyrene beads coated with either DENV2 or a mix of DENV1, 3 and 4 were used to deplete DENV-binding antibodies from DENV2 primary immune sera, DT001 (A,B,C,D,E and F) and DT 110 (G,H,I,J,K and L). Following depletion of DENV-binding antibodies, sera was tested for binding (A, D, G and J) and neutralization of DENV1-4 (B, C, E, F, H, I,K and L). Error bars indicate Standard Error of the Mean (SEM).
(DOCX)

S2 Fig Binding and neutralization properties of repeat infection DENV-immune human sera following depletion of DENV-binding antibodies. Polystyrene beads coated with either DENV2 or a mix of DENV1, 3 and 4 were used to deplete DENV-binding antibodies from repeat infection DENV-immune sera, DT000, DT130, DT121, DT027 and DT025. Following depletion of DENV-binding antibodies, sera was tested for binding (A, D, G, J, M, P, S, V, Y and BB) and neutralization (B, C, E, F, H, I, K, L, N, O, Q, R, T, U, W, X, Z, AA, CC and DD) of DENV1-4. Error bars indicate Standard Error of the Mean (SEM).
(DOCX)

S3 Fig Binding and neutralization properties of post-second infection DENV-immune human sera following depletion of DENV-binding antibodies. Subjects 985 **(A—D)** and 3428 **(I—L)** experienced DENV2 → DENV3 infections. Subject 2934 **(E-H)** experienced DENV1 → DENV3 infections. Polystyrene beads coated with either the DENV serotype of the first or second infection were used to deplete specific populations of DENV-binding antibodies from sera collected after the second infection. Following depletion, sera was tested for binding **(A, E and I)** and neutralization **(B, C, D, F, G, H, J, K and L)** of DENV1-4.
(DOCX)

S4 Fig Binding and neutralization properties of primary infection DENV2-immune human sera following depletion of DENV2 rE- binding antibodies. Beads conjugated to DENV2 rE were used to deplete rE-binding antibodies in human immune sera. The depleted sera were assessed for binding to rE **(A, E)** and whole virions **(B, F)** from DENV1-4 as well as neutralization **(C, D, G and H)** of DENV1-4.
(DOCX)

S5 Fig Binding and neutralization properties of repeat infection DENV-immune human sera following depletion of DENV2 rE-binding antibodies. Beads conjugated to DENV2 rE were used to deplete rE-binding antibodies in human immune sera. The depleted sera were assessed for binding to rE **(A, E, I, M, Q)** and whole virions **(B, F, J, N, R)** from DENV1-4 as well as neutralization **(C, D, G, H, K, L, O, P, S, T)** of DENV1-4. Error bars indicate Standard Error of the Mean (SEM).
(DOCX)
