# Supplementary material for: Dissecting the human serum antibody response to secondary dengue virus infections
Source: PLoS Negl Trop Dis. 2017 May 15;11(5):e0005554. doi: 10.1371/journal.pntd.0005554 (PMC5444852; doi:10.1371/journal.pntd.0005554)
Supplement: S1 Fig — Polystyrene beads coated with either DENV2 or a mix of DENV1, 3 and 4 were used to deplete DENV-binding antibodies from DENV2 primary immune sera, DT001 (A,B,C,D,E and F) and DT 110 (G,H,I,J,K and L). Following depletion of DENV-binding antibodies, sera was tested for binding (A, D, G and J) and neutralization of DENV1-4 (B, C, E, F, H, I,K and L). Error bars indicate Standard Error of the Mean (SEM). (DOCX) [file pntd.0005554.s001.docx]

**S1 Fig. Binding and neutralization properties of primary infection DENV2-immune human sera following depletion of DENV-binding antibodies.** Polystyrene beads coated with either DENV2 or a mix of DENV1, 3 and 4 were used to deplete DENV-binding antibodies from DENV2 primary immune sera, DT001 (A,B,C,D,E and F) and DT 110 (G,H,I,J,K and L). Following depletion of DENV-binding antibodies, sera was tested for binding (A, D, G and J) and neutralization of DENV1-4 (B, C, E, F, H, I,K and L). Error bars indicate Standard Error of the Mean (SEM).
