# Supplementary material for: Dissecting the human serum antibody response to secondary dengue virus infections
Source: PLoS Negl Trop Dis. 2017 May 15;11(5):e0005554. doi: 10.1371/journal.pntd.0005554 (PMC5444852; doi:10.1371/journal.pntd.0005554)
Supplement: S2 Fig — Polystyrene beads coated with either DENV2 or a mix of DENV1, 3 and 4 were used to deplete DENV-binding antibodies from repeat infection DENV-immune sera, DT000, DT130, DT121, DT027 and DT025. Following depletion of DENV-binding antibodies, sera was tested for binding (A, D, G, J, M, P, S, V, Y and BB) and neutralization (B, C, E, F, H, I, K, L, N, O, Q, R, T, U, W, X, Z, AA, CC and DD) of DENV1-4. Error bars indicate Standard Error of the Mean (SEM). (DOCX) [file pntd.0005554.s002.docx]

Figure S2

Figure S2 cont.

**S2 Fig. Binding and neutralization properties of repeat infection DENV-immune human sera following depletion of DENV-binding antibodies.** Polystyrene beads coated with either DENV2 or a mix of DENV1, 3 and 4 were used to deplete DENV-binding antibodies from repeat infection DENV-immune sera, DT000, DT130, DT121, DT027 and DT025. Following depletion of DENV-binding antibodies, sera was tested for binding (A, D, G, J, M, P, S, V, Y and BB) and neutralization (B, C, E, F, H, I, K, L, N, O, Q, R, T, U, W, X, Z, AA, CC and DD) of DENV1-4. Error bars indicate Standard Error of the Mean (SEM).
