# Supplementary material for: Dissecting the human serum antibody response to secondary dengue virus infections
Source: PLoS Negl Trop Dis. 2017 May 15;11(5):e0005554. doi: 10.1371/journal.pntd.0005554 (PMC5444852; doi:10.1371/journal.pntd.0005554)
Supplement: S3 Fig — Subjects 985 (A—D) and 3428 (I—L) experienced DENV2 → DENV3 infections. Subject 2934 (E-H) experienced DENV1 → DENV3 infections. Polystyrene beads coated with either the DENV serotype of the first or second infection were used to deplete specific populations of DENV-binding antibodies from sera collected after the second infection. Following depletion, sera was tested for binding (A, E and I) and neutralization (B, C, D, F, G, H, J, K and L) of DENV1-4. (DOCX) [file pntd.0005554.s003.docx]

**S3 Fig. Binding and neutralization properties of post-second infection DENV-immune human sera following depletion of DENV-binding antibodies.** Subjects 985 **(A–D)** and 3428 **(I–L)** experienced DENV2 🡪 DENV3 infections. Subject 2934 **(E-H)** experienced DENV1 🡪 DENV3 infections. Polystyrene beads coated with either the DENV serotype of the first or second infection were used to deplete specific populations of DENV-binding antibodies from sera collected after the second infection. Following depletion, sera was tested for binding **(A, E and I)** and neutralization **(B, C, D, F, G, H, J, K and L)** of DENV1-4.
