# Supplementary material for: Dissecting the human serum antibody response to secondary dengue virus infections
Source: PLoS Negl Trop Dis. 2017 May 15;11(5):e0005554. doi: 10.1371/journal.pntd.0005554 (PMC5444852; doi:10.1371/journal.pntd.0005554)
Supplement: S4 Fig — Beads conjugated to DENV2 rE were used to deplete rE-binding antibodies in human immune sera. The depleted sera were assessed for binding to rE (A, E) and whole virions (B, F) from DENV1-4 as well as neutralization (C, D, G and H) of DENV1-4. (DOCX) [file pntd.0005554.s004.docx]

**S4 Fig. Binding and neutralization properties of primary infection DENV2-immune human sera following depletion of DENV2 rE- binding antibodies.** Beads conjugated to DENV2 rE were used to deplete rE-binding antibodies in human immune sera. The depleted sera were assessed for binding to rE **(A, E)** and whole virions **(B, F)** from DENV1-4 as well as neutralization **(C, D, G and H)** of DENV1-4.
