# Supplementary material for: Dissecting the human serum antibody response to secondary dengue virus infections
Source: PLoS Negl Trop Dis. 2017 May 15;11(5):e0005554. doi: 10.1371/journal.pntd.0005554 (PMC5444852; doi:10.1371/journal.pntd.0005554)
Supplement: S5 Fig — Beads conjugated to DENV2 rE were used to deplete rE-binding antibodies in human immune sera. The depleted sera were assessed for binding to rE (A, E, I, M, Q) and whole virions (B, F, J, N, R) from DENV1-4 as well as neutralization (C, D, G, H, K, L, O, P, S, T) of DENV1-4. Error bars indicate Standard Error of the Mean (SEM). (DOCX) [file pntd.0005554.s005.docx]

**Figure S5**

**Figure S5 Cont.**

**S5 Fig. Binding and neutralization properties of repeat infection DENV-immune human sera following depletion of DENV2 rE-binding antibodies.** Beads conjugated to DENV2 rE were used to deplete rE-binding antibodies in human immune sera. The depleted sera were assessed for binding to rE **(A, E, I, M, Q)** and whole virions **(B, F, J, N, R)** from DENV1-4 as well as neutralization **(C, D, G, H, K, L, O, P, S, T)** of DENV1-4. Error bars indicate Standard Error of the Mean (SEM).
